# Surprising astigmatism hypercorrection after corneal ring segments implantation in keratoconus treatment after 8 years of follow up

**DOI:** 10.22336/rjo.2021.16

**Published:** 2021

**Authors:** Guilherme Malta Pio, Frederico Malta Pio, Alessandra Mariano Caldeira Coelho, Carolina Serpa Braga, Anna Flávia Ribeiro Pereira, Caroline Alves Cotrim, Frederico Bicalho Dias Silva

**Affiliations:** * Department of Ophthalmology, Instituto de Olhos Ciências Médicas, Belo Horizonte/MG, Brazil; ** Department of Surgical Retina, Hospital São Geraldo - HC/UFMG, Belo Horizonte/MG, Brazil; *** Department of Ophthalmology, Santa Casa de Misericórdia, Belo Horizonte/MG, Brazil; **** Department of Cornea, Hospital São Geraldo - HC/UFMG, Belo Horizonte/MG, Brazil

**Keywords:** corneal ring, keratometric astigmatism, keratoconus, keratometry, corneal implant

## Abstract

**Objective:** To report a case of hypercorrection of astigmatism (Cyl) after implantation of 2 segments of short arch ring for keratoconus treatment and to describe its replacement by long arch segment.

**Methods:** This is a case report of a patient with keratoconus and no adaptation to glasses or contact lenses, who was implanted 2 ring segments: upper nasal (155º/ 200μm) and inferior temporal (155º/ 250μm).

**Results:** First postoperative month: CVA = 20/ 50 (-10.50-2.50x135°) and SimK K1 = 48.4x143° and K2 = 51.2x53° (Cyl 2,8D). In the 3rd year: CVA 20/ 30 (-6.00-2.50x135º), with inversion of the axes: K1 = 49,5x60º and K2 = 52,0x150º (Cyl 2,6D). The hypercorrection increased up to the 8th year: CVA = 20/40 (-4,50-6,00x75º) and SimK 47,8x51º/ 60,4x141º (Cyl 12,6D). The 2 segments were replaced for a single segment (320º/ 300μm) and after 1 month: CVA = 20/ 25 (-5,75 spherical) with SimK 46,8x38º/ 48,9x128º (Cyl 2,1D).

**Conclusion:** The ring aims to flatten the most curved meridian, but surpassing the previous value induces astigmatism in the opposite meridian. The hypercorrection of the 2 short segments must occur due to its movement of the extremities, which does not occur with the single long arc segment (≥ 300º).

**Abbreviations:** CVA = Corrected visual acuity, SimK = Simulated keratometry, LE = Left eye, RE = Right eye

## Introduction

Keratoconus is a hereditary pathology, usually bilateral, that generates low vision by inducing myopia and astigmatism, as well as altering the regularity and transparency of the cornea [**1**]. Its incidence in the population is controversial in literature, ranging from 1:500 to 1:2000 individuals [**2**].

In most cases, it begins in adolescence and can develop until approximately 35 years of age. At first, the simple use of glasses is enough to give back a good vision to the patient. With evolution, the glasses are no longer of value and the rigid contact lenses become the only resource capable of producing clear images. When corneal astigmatism becomes even more important, it becomes impracticable to adapt these lenses as they become very uncomfortable or fall out of the eyes frequently. In these phases, the only effective options are the surgical procedures, among them the implant of the corneal ring segment and the cornea transplant [**3**].

In 1936, the Spanish ophthalmologist Ramón Castroviejo Briones successfully performed the first corneal transplant for the treatment of keratoconus [**4**]. In 1949, José Ignácio Barraquer began the use of intracorneal implants with the purpose of correcting myopia [**5**]. Their findings ground the principles for selecting the appropriate corneal ring segment to reshape each cornea. It is known as the Barraquer’s Law: “Whenever a tissue is added to the periphery or removed from the center of the cornea, a corresponding flattening is obtained”. Following this orientation, it is possible to conclude that the greater the protrusion and the corneal irregularity (the more advanced the keratoconus), the thicker is the device to be implanted [**6**].

**Fig. 1:**
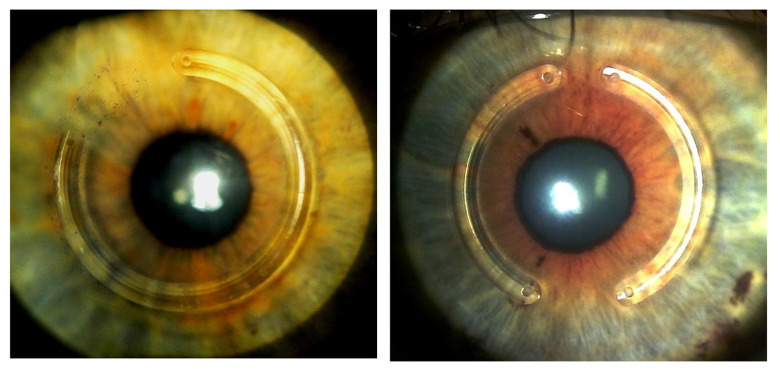
Left: Postoperative aspect of an eye that was submitted to the implant of 1 segment of corneal ring with 300º of arch length. Right: aspect of the implant of 2 segments of ring cornea, each with 155º of length of arc. Source: author

Inspired by Barraquer’s ideas, the Brazilian ophthalmologist Paulo Ferrara de Almeida Cunha developed a corneal implant that became known as the “Ferrara’s Ring” [**7**]. During the early development of annular corneal implants in Brazil (late 20th century), models with a large arch length (355° arc) were tested for myopia correction. However, the technical difficulty for its implantation was one of the reasons that led to its abandonment and replacement by 2 smaller ring segments (160° arc each) – **Fig. 1** [**8**].

In 1995, Paulo Ferrara began using these devices for the treatment of irregular corneas, among which, those with keratoconus. Later works by Paulo Ferrara, Coscarelli and others demonstrated the relevance of the correction of corneal astigmatism in irregular corneas, to allow a good adaptation of contact lenses or promote an improvement of vision with glasses [**9**].

Since then, more and more attention has been given to the need to correct corneal astigmatism for the visual rehabilitation of the patient with keratoconus [**8**]. Gradually, this new therapeutic modality was refined by better surgical techniques and equipment, improving effectiveness and reproducibility, and attracting the interest of doctors from various parts of the world [**9**].

With the advent of the femtosecond laser (principle of the 21st century), it has become much easier to implant large ring-length segments. Thus, we had the re-launch of these great devices around 2010 [**9**].

Studies are being conducted to see whether these large single segments (usually 300 or 320- or 325-degrees arc) can produce better results than the 2 smaller segments used in the traditional technique [**10**].

It is important to emphasize that the surgical technique for the introduction of these new devices (300 or 320 or 325 degrees of arc) is much more difficult and time consuming than the traditional segment implant technique. Thus, the acceptance of these new devices by physicians depends on the overcoming of two barriers: the increase in the difficulty of the surgical technique and the absence of proof of its greater effectiveness in the visual rehabilitation of the patient with keratoconus [**11**].

The objective of this work was to demonstrate a case of keratoconus that was initially treated with a double corneal ring segment implant and evolved with a hypercorrection of astigmatism. To correct this complication, it was necessary to explant the short segments and replace them by a single-segment implant of 320 degrees.

## Materials and methods - case report

A 28-year-old female patient with keratoconus diagnosed 5 years before the beginning of the follow-up and family history of two uncles with keratoconus (one of them undergoing corneal transplantation) reported poor adaptation to glasses and rigid contact lenses. The examination revealed the following: right eye with corrected visual acuity (CVA) of 20/50 (Snellen) (plane-4,00x45°) and simulated keratometry (SimK) of the topography with K1 = 46,1x51º and K2 = 51,6x141º (Cyl 5,5D); left eye (LE) with CVA of 20/ 100 (-19.00-7.50x170°) and SimK of topography with K1 = 50,3x134º and K2 = 57,5x44º (Cyl 7,2D); biomicroscopy of both eyes with Vogt striations, more intense in LE; normal fundoscopy and intraocular. A ring was initially indicated for the LE and, according to the manufacturer’s instructions (Cornealring - Visiontech Ltda), an implant with 2 short segments, superior nasal ring of 155º/ 200μm and inferior temporal of 155º/ 250 μm was chosen.

## Results

The surgical planning was done according to the corneal map of thickness, obtained from the pachymetry. The procedure was performed without intercurrences, with manual technique and the devices were inserted in good depth in the most curved meridian.

There was good visual and keratometric results by the end of the 1st postoperative month: LE with CVA = 20/ 50 (-10.50-2.50x135°) and SimK with K1 = 48,40x143º and K2 = 51,20x53º (Cyl 2,8D). At biomicroscopy, the ring segments were in good depth; slope of 43 degrees; the upper nasal segment moved to near the incision, maintaining approximately 0.5 mm from the incision, which remained closed.

In the 3rd year after implantation, visual acuity and keratometry were even better: LE with CVA = 20/ 30 (-6.00-2.50x135º). However, the topography showed a slight overcorrection and inversion of the Cyl axis, with K1 = 49,50x60º and K2 = 52,00x150º (Cyl 2,6D). This hypercorrection presented progressive increase and in the 8th year, the patient’s LE presented the following CVA = 20/ 40 (-4.50-6.00x75º) and SimK K1 = 47,80x51º and K2 = 60,40x141º (Cyl 12,6D).

**Fig. 2:**
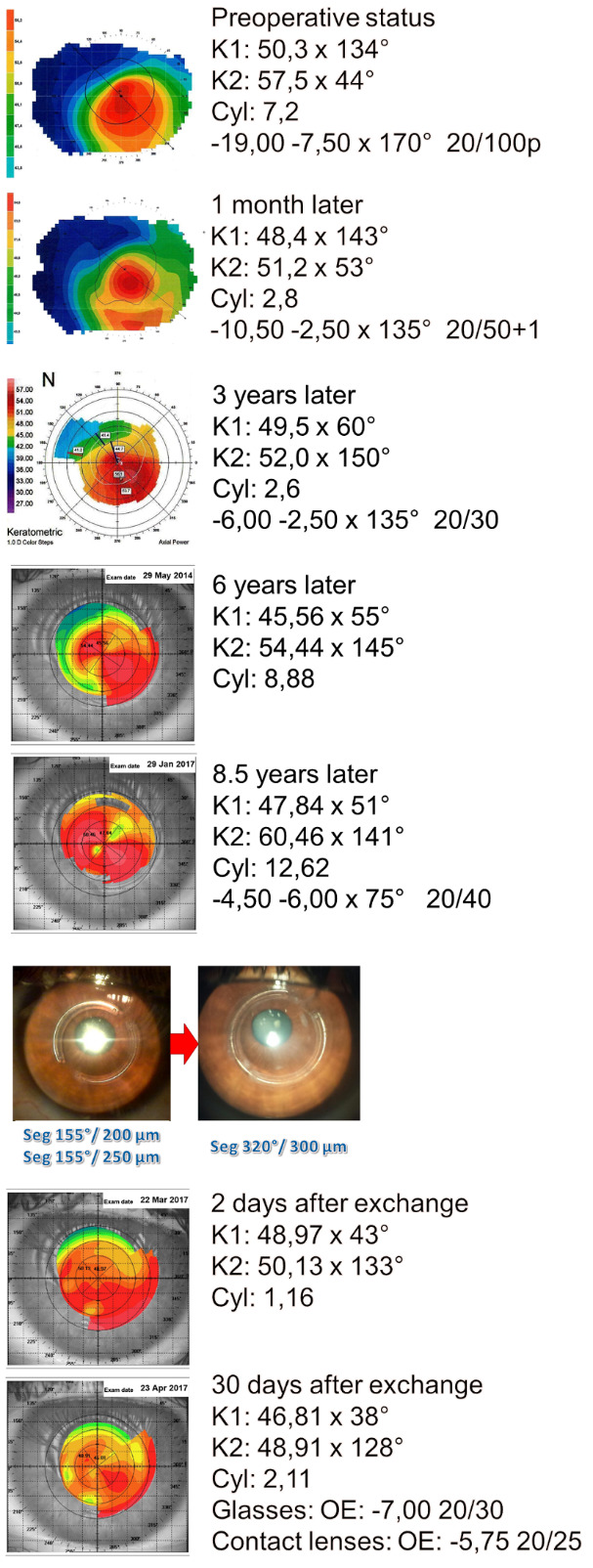
Refractive and topographic evolution of the left eye, from the time of admission to the moment after the rings were replaced, during the 8-year follow-up. Source: author.

Intervention was indicated due to this hypercorrection and the 2 ring segments were replaced by a single one of 320º/ 300μm segment (Ferrara’s Ring - AJL). Two days after the replacement, the keratometry SimK presented K1 = 48.9x43º and K2 = 50.1x133º (Cyl 1,16D) and after 1 month, the CVA was 20/ 25 (-5.75 spherical) and SimK K1 = 46.8x38° and K2 = 48.9x128° (Cyl 2,1D). **Fig. 2** summarizes the entire refractive and keratometric evolution from the moment of admission until after the replacement of the rings.

Regarding the evolution of the right eye (RE), a ring implant was indicated three years after the patient’s admission. At that time, the patient presented CVA = 20/ 50 (plane-4,00x45º) and keratometry SimK with K1 = 46,10x51º and K2 = 51,60x141º (Cyl 5,50D). According to the manufacturer’s guidance at the time (Cornealring - Visiontech Ltda), the implant of 2 ring segments was chosen: CR5/ 155º/ 200 micra and CR5/ 155º/ 150 micra. The evolution of the right eye is summarized in **Table 1**.

**Tabel 1:** Refractive and keratometric evolution of the right eye after intracorneal ring segment implantation

Follow up	SimK	Cyl	Refraction	CVA
Immediate preoperative	K1 = 46,10x51º	5,50D	plane-4,00x45º	20/ 50
	K2 = 51,60x141º			
2 months	K1 = 48,90x118º	2,10D	-1,50-2,00x60º	20/ 50
	K2 = 51,00x28º			
3 years	K1 = 50,75x110º	1,43D	-5,50-2,50x35º	20/ 40
	K2 = 52,18x20º			
4 years	K1 = 50,95x104º	1,71D	-8,50-3,00x50º	20/ 30
	K2 = 52,66x14º			
6 years	K1 = 52,44x97º	2,38D	-11,50-3,00x60º	20/ 30
	K2 = 54,82x7º			

The analysis of the RE’s evolution showed that there was also an inversion of the axis of astigmatism in the immediate postoperative period and, over the years, a progressive increase of the corneal curvature with an increasing refractive myopia. Still, in the sixth year, the patient was satisfied with the vision, wearing glasses and rigid contact lenses, and at that moment, right eye’s rings have not been replaced.

## Discussion

For the correction of astigmatism, ring surgery should promote a flattening of the more curved meridian (K2) so that it approached the curvature value of the flatter meridian (K1). Thus, if the astigmatic correction was greater than the preoperative astigmatism, we would have had the induction of astigmatism in the meridian opposite to the preexisting one. This means that excessive corrections (hypercorrections) are as undesirable as hypocorrections [**11**].

**Fig. 3:**
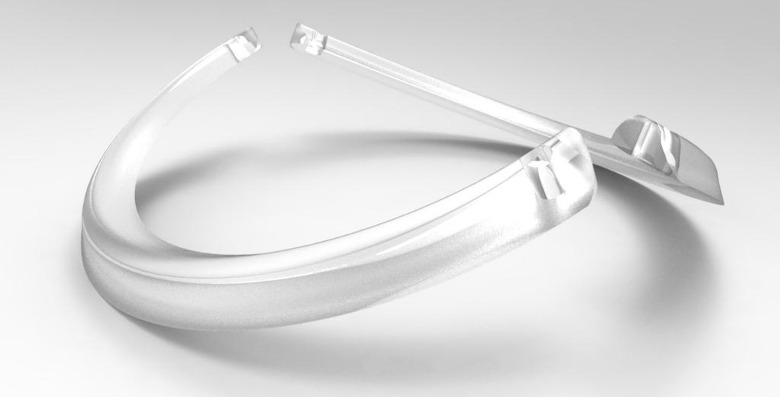
Possible lifting movement of the extremities of the 155° arc segments, which may explain the occurrence of cases of hypercorrection of astigmatism

Hypercorrection cases are more frequent in double-segment implants. This can be explained by the movement of the extremities of these segments upwards, creating an excessive flattening of the meridian where they were positioned – **Fig. 3**. The single segment of 300 degrees of arc does not present this inclination with elevation of its extremities, which can be the explanation for the non-occurrence of cases of significant overcorrection [**12**].

In the case described here, the implant of two ring segments in the left eye occurred without intercurrences and with good initial results. However, as of the third postoperative year, there was a progressive overcorrection of astigmatism with reversal of the cylinder axis. Replacement of the two segments by single long ring segment corrected excessive flattening.

Similarly, after the surgical intervention using two ring segments, the right eye evolved with inversion of the axis of astigmatism and with progressive increase of corneal curvature (increased myopia). According to the presented data, the approach of this eye should probably follow the same reasoning, with the explant of the double segment and replacement by a single long segment implant.

It is important to note that implant surgery of the 300º arc segment involves a greater operative difficulty. It happens because, for each fraction of the segment entering the corneal tunnel, there is a greater adhesion and more strength must be made for its displacement. However, this force cannot be exaggerated to prevent the ring from breaking, and it is not prudent to insist upon realizing when the implant advance has ceased. In these cases, the implant should be removed and a new tunneling (wider than the previous) should be performed or lubricant may be used within the tunnel to promote this displacement (usually viscoelastic substances as used in cataract surgery). This procedure should be repeated as many times as necessary until the complete introduction of the segment is achieved with relative ease.

## Conclusion

The importance of this case report is to attempt to instigate research that may elucidate the extent to which the implant of a single 300 degrees segment of a corneal ring is more effective in the vector correction of keratometric astigmatism than the traditional 155-degree segments.

A favorable result to the use of the large arch segments could justify its adoption by the medical practice, even though there is a greater technical difficulty inherent in its use. If its superiority is proven, a revision of the surgical technique is necessary in order to make it less difficult. New technologies can be quite useful to facilitate this type of surgery, especially the “femtosecond laser” that has been bringing a great contribution in the construction of more regular and perfectly centralized corneal tunnels.

It will be interesting to deepen this study, especially with large samples and with longer follow-up time, in order to observe cases like this that evolve on the long term.

**Conflict of Interest**

Authors state no conflict of interest.

**Informed Consent and Human and Animal Rights statements**

Informed consent has been obtained from all individuals included in this study.

**Authorization for the use of human subjects**

Ethical approval: The research related to human use complies with all the relevant national regulations, institutional policies, is in accordance with the tenets of the Helsinki Declaration, and has been approved by the Ethics Committee of Instituto de Olhos Ciências Médicas, Belo Horizonte/ MG, Brazil.

**Acknowledgements**

None.

**Sources of Funding**

The authors declare that their research has not been funded by any entity.

**Disclosures**

None.
